# Does our Mycobacteriome Influence COVID-19 Morbidity and Lethality?

**DOI:** 10.3389/fmicb.2021.589165

**Published:** 2021-02-04

**Authors:** Armando Acosta, Luis Fonte, Maria E. Sarmiento, Mohd Nor Norazmi

**Affiliations:** ^1^School of Health Sciences, Universiti Sains Malaysia, Kelantan, Malaysia; ^2^Department of Parasitology, Institute of Tropical Medicine “Pedro Kourí”, Havana, Cuba

**Keywords:** mycobacteriome, COVID-19, bacille calmette-guérin vaccine, non-tuberculous mycobacteria, Latent TB infections

COVID-19, caused by SARS-CoV-2, can be asymptomatic, manifest as mild clinical symptoms, or go to pneumonia and multi-organ failure that can be fatal. This range of clinical manifestations is a consequence of the activation of the innate and adaptive immune responses which seem to contain viral replication and lead to recovery and, in the more unfavorable sequelae, can trigger an intense inflammatory reaction, leading to serious clinical complications, and death (Wang et al., 2020).

Despite the concerns on the health care infrastructures and the high proportion of people living in poverty and overcrowded urban settings, COVID-19 in Sub-Saharan Africa (SSA) has not resulted in the anticipated huge number of confirmed cases and deaths (World Health Organization, 2020).

A combination of several factors has been put forward to explain the unexpectedly lower rates of incidence and lethality—non-extensive diagnostic testing, younger age group, host genetic background, SARS-CoV-2 mutations, higher temperature, environmentally less-favorable for viral replication, endemic infections, Bacillus Calmette-Guérin (BCG) vaccination policy, and microbiome, among others (Fonte et al., 2020; Janda et al., 2020; Khatiwada and Subedi, 2020; Mbow et al., 2020).

One of the components of the human microbiome is the mycobacteriome, composed by mycobacteria (Macovei et al., 2015). The term “mycobacteriome” was coined associated with non-tuberculous mycobacteria (NTM), defined by the authors as “non-tuberculous mycobacteriome” (Macovei et al., 2015), but considering the presence of BCG decades after vaccination (Armbruster et al., 1990; Van Deutekom et al., 1996; Talbot et al., 1997) and the evidence of *Mycobacterium tuberculosis* (Mtb) infection in healthy individuals [latent tuberculosis infection (LTBI)] (Barrios-Payán et al., 2012; Mayito et al., 2019; Mehaffy et al., 2020), we consider that the concept of mycobacteriome could be expanded including NTM, BCG, and Mtb.

Many factors influence microbiome composition, such as genetic background, environmental and socioeconomic conditions, geographical region, lifestyles, and alimentary habits, among others. BCG vaccination, the presence of LTBI and environmental NTM, in low- and medium-income countries, such as those that belong to SSA, may present a distinctive mycobacteriome compared to those of high-income countries. This distinct mycobacteriome has been suggested to influence the susceptibility to infections, allergy, and autoimmunity (Sewell et al., 2002; Obihara et al., 2006; Nemeth et al., 2019).

Recent reports suggested the possibility that BCG vaccination has a positive effect on the prevention of COVID-19 and its lethality (Escobar et al., 2020; Netea et al., 2020). The explanation of this phenomenon has been based on the “trained immunity” mechanism, induced by BCG vaccination, which has been demonstrated for different infectious diseases in children and in experimental viral infection in humans (Arts et al., 2018; Netea et al., 2020). It has been also suggested that the protective effect of BCG could be mediated by specific responses against shared epitopes between SARS-CoV-2 and BCG (Reche, 2020; Strongin et al., 2020; Tomita et al., 2020). However, some authors argued that the protective effect of BCG against COVID-19 is not clearly demonstrated, and after considering potential confounding factors, such as SARS-CoV-2 testing rate, the protective effect vanished (Hensel et al., 2020; Redelman-Sidi, 2020; Shivendu et al., 2020; Wassenaar et al., 2020).

The impact of the mycobacteriome on the protection against SARS-CoV-2 infection and its related disease could probably be explained beyond BCG vaccination. The potential impact of LTBI, present in one-quarter of the human population (Houben and Dodd, 2016), with high prevalence in regions where BCG immunization is implemented, should be also considered. It has been suggested that the positivity of Tuberculin Skin Test (TST) and/or Interferon Gamma Release Assays correlate better with lower incidence and lethality of COVID-19 than with BCG vaccination (Singh, 2020). However, a small study with 36 positive SARS-CoV-2 cases suggested a detrimental effect of LTBI and TB on the COVID-19 evolution (Liu et al., 2020).

LTBI triggers an increase in M1 macrophage presence, both in mice and humans, which is compatible with the activation of innate immune responses, as has been described in the context of “trained immunity” (Nemeth et al., 2019; Netea et al., 2020). The same results have also been reported with vaccine candidates based on attenuated Mtb strains in mice (Tarancón et al., 2020). It has been reported that LTBI prevents asthma and abrogates eosinophilopoiesis in an experimental model (Tarancón et al., 2019). LTBI was associated with protection against allergy in TB endemic areas (Obihara et al., 2006). The same study found that the intensity of the TST reactivity was inversely correlated with the development of allergic reactions, which suggested a role of a biased Th1-type response with downregulation of Th2 responses (Obihara et al., 2006). These studies support the opinion that LTBI, through mechanisms of innate immunity, produces a bystander effect on acquired immune responses, which could generate a protective environment against heterologous microorganisms, including SARS-CoV-2.

Other members of the mycobacteriome with potential influence on COVID-19 severity are NTM, which, due to their worldwide distribution, are also present in areas covered by BCG vaccination. NTM have been associated with the production of a regulatory environment mediated by transforming growth factor-β (TGF-β) and interleukin-10 (IL-10), induction of T regulatory cells, and inhibition of neutrophil infiltration (Zuany-Amorim et al., 2002a,b). Such an environment could provide protection against the severe forms of COVID-19, inhibiting the uncontrolled inflammation resulting in the so-called “cytokine storm.”

In summary, the lower incidence and lethality rates of COVID-19 registered in SSA countries could be a consequence, among other factors, of a blend of different immunological mechanisms, some of them induced by members of the mycobacteriome: BCG and LTBI affecting SARS-CoV-2 multiplication through trained immunity and cross-reactive immune responses, and NTM limiting the pathological inflammation triggered by the host immune response against the virus, through the induction of a regulatory environment.

## Author Contributions

All authors listed have made a substantial, direct and intellectual contribution to the work, and approved it for publication.

## Conflict of Interest

The authors declare that the research was conducted in the absence of any commercial or financial relationships that could be construed as a potential conflict of interest.
